# Prediction of Radix Astragali Immunomodulatory Effect of CD80 Expression from Chromatograms by Quantitative Pattern-Activity Relationship 

**DOI:** 10.1155/2017/3923865

**Published:** 2017-02-28

**Authors:** Michelle Chun-har Ng, Tsui-yan Lau, Kei Fan, Qing-song Xu, Josiah Poon, Simon K. Poon, Mary K. Lam, Foo-tim Chau, Daniel Man-Yuen Sze

**Affiliations:** ^1^Department of Health Technology and Informatics, The Hong Kong Polytechnic University, Hung Hom, Hong Kong; ^2^Department of Applied Biology and Chemical Technology, The Hong Kong Polytechnic University, Hung Hom, Hong Kong; ^3^School of Mathematics and Statistics, Central South University, Changsha 410083, China; ^4^School of Information Technologies, The University of Sydney, Lidcombe, NSW, Australia; ^5^Faculty of Health, University of Technology Sydney, Ultimo, NSW, Australia; ^6^School of Health and Biomedical Sciences, RMIT University, Melbourne, VIC, Australia

## Abstract

The current use of a single chemical component as the representative quality control marker of herbal food supplement is inadequate. In this CD80-Quantitative-Pattern-Activity-Relationship (QPAR) study, we built a bioactivity predictive model that can be applicable for complex mixtures. Through integrating the chemical fingerprinting profiles of the immunomodulating herb* Radix Astragali* (RA) extracts, and their related biological data of immunological marker CD80 expression on dendritic cells, a chemometric model using the Elastic Net Partial Least Square (EN-PLS) algorithm was established. The EN-PLS algorithm increased the biological predictive capability with lower value of RMSEP (11.66) and higher values of *R*_*p*_^2^ (0.55) when compared to the standard PLS model. This CD80-QPAR platform provides a useful predictive model for unknown RA extract's bioactivities using the chemical fingerprint inputs. Furthermore, this bioactivity prediction platform facilitates identification of key bioactivity-related chemical components within complex mixtures for future drug discovery and understanding of the batch-to-batch consistency for quality clinical trials.

## 1. Introduction

A large pool of medicinal plants from Chinese herbal medicines (CHM) has a long historical clinical practice for more than 2000 years ago. However, the underlying mechanisms of action of the CHM remain largely unknown except the few examples of taxol [1] for anticancer, artesunate [2] for malaria treatment, and arsenic trioxide [3] for leukemia treatment. While these three herbal derived single compounds are responsible for the effective therapies, however, for most of the other clinically useful CHM, the mechanisms of action have been considered as that of “multicompound multitarget.” The use of herbal formula by combining a few herbs based on the Chinese medicine theory further adds to this complexity. Thus, there exists a wide range of possible chemical compounds in each single herb or complex formula that may contribute to the clinical efficacy, but this crucial information is basically unknown at the moment. This lack of understanding of the active compounds and their targets in turn makes the quality control aspect of ensuring the batch-to-batch consistency of CHM difficult if not impossible.

Up to now, a CHM product PHY906 which is undergoing phase 2 clinical trial and being marketed as an adjuvant to chemotherapy attempted to address the batch-to-batch consistency issue [4, 5]. The researchers established a platform of “Phytoceutica” to address the similarity index of products of different batches for both of their chemical fingerprinting using liquid chromatography-mass spectrometry (LCMS) and the biological fingerprinting by microarray profiling. While this platform suffices for the purpose of quality control, it is not powerful enough to help the identification of compounds that are actually related to the mechanism of actions, such as reducing chemotherapy-induced gastrointestinal toxicity in mice [4] and the clinically favorable outcomes in cancer [5]. Hence, there is an increasing attention in research development to evaluate the mixtures of compounds from the CHM extracts as a whole with the bioactivity for developing modern drugs.

The conventional component-based quality control approach may overestimate the therapeutic value of some highly representative components while the minor components are ignored for their active roles or masked in the crude extract [6]. Accordingly selecting only the major chemical components as the standard markers is not adequate to explain the total therapeutic effect of the CHM. More importantly, as different constituents may contribute to different therapeutic activities, therefore a single CHM may possess multiple therapeutic activities. Understanding the quantitative relationship between the multiple chemical constituents of a single CHM with the corresponding bioactivity is becoming imperative.

In the last few years, different international research teams have attempted to address this quality control of herbal medicines issue by both the chemical and biological fingerprinting approaches. For instance, in China, Yan and his colleagues [7] studied 28 samples of Radix Tinosporae for analgesic bioactivities on mice. Chen and his colleagues used 32 combinations of 5-herb CHM mixture including RA and studied antiplatelet in SD rats [8]. Another research group of Jiang is based on 31 batches of curcuma volatile oil to study antitumor in vitro effects [9]. In Belgium, Tistaert and his colleagues reported similar approach using 39 Mallotus extracts and examined the related cytotoxicity [10]. Also in Singapore, Ching and his colleagues used 6 different solvent systems to extract the A.* elliptica* leaves and studied the corresponding antiplatelet activities [11].

Our laboratory has also developed comprehensive methods with multicomponent quantification such as pattern-based approaches through chemometric data processing techniques that have been used for the identification of contributing elements within a mixture [12, 13]. In this study, we adopted and expanded our laboratory's chemometric methodology named Quantitative-Pattern-Activity-Relationship (QPAR) to study an immunomodulatory herbal medicines, Radix Astragali (RA, or commonly known as Huangqi) [12].

QPAR is a computer-assisted platform based on the application of statistics and data analytical methods for model development [12]. It simply colligates the extract of a single herb as chemical fingerprint with the corresponding biological activity. A statistical mathematical model is then built for revealing the valuable information of CHM related to the corresponding bioactivity. These developed models can also be used for predicting the biological activity of a HM based solely on its intact chemical fingerprint.

It is well known that RA is one of the most widely used CHM for the enhancement of “qi” based on Chinese medicine theory. About its related mechanisms of action, a few publications have demonstrated that RA is related to the increase of both humoral immunity [14] and cellular immunity in our body [15, 16] or immunomodulatory as a whole [17–20]. RA has also been shown to exert an anticarcinogenic effect in carcinogen-treated mice through activation of cytotoxic activity and the production of cytokines [21]. We have previously published that dendritic cells (DCs), as the most important professional antigen presenting cells in anticancer immunity, have been found to be defective in cancer patients [22, 23]. Furthermore, this defectiveness increased when cancer progressed to more advanced stage. It is known that CD80 is the most important costimulatory molecules on the surface of DCs to provide the crucial second signal for the proper stimulation of cancer antigen-specific naive T cells.

Therefore, in this project, we harness the knowledge of the prior QPAR methodology and the CD80 flow cytometric bioactivity platform. By producing more than 70 crude extracts of RA of varied components, we aimed to build a CD80-QPAR model of RA. With this model, we can demonstrate the model's predictability with the chromatogram alone of any new RA preparations as an input, and the corresponding bioactivity can be accurately predicted. This knowledge is important for the determination of the levels of bioactivity-related quality control chemical markers in herbal extracts to be used in clinical trials.

## 2. Materials and Methods

### 2.1. Preparation of Radix Astragali (RA) Extracts, Reagents, and Reference Compound

Three batches of raw RA (RA-A, RA-B, and RA-C) were used to prepare 72 extracts in total (24 extracts each) according to a modified extraction method based on the Chinese Pharmacopoeia. Briefly, 4 g raw herb was preimmersing with bidistilled water (100, 150, 200, and 250 mL) for 12 hr and refluxed for 0, 1, 2, 3, and 4 hrs. The mixtures were then filtered and concentrated under a rotary evaporator (Brand, Germany). RA extracts were finally obtained after lyophilisation. Each extract was stored under low humidity condition and was kept for biological assay within 3 months. All the extracts before chromatographic analysis and biological assay were filtered under 0.2 *μ*m filter. Bidistilled water was produced in-house by Milli-Q® Advantage A10 water purification systems (Millipore; USA) and filtered with 0.22 *μ*m Millipak®. All other chemicals and reagents used were of analytical grade unless indicated otherwise.

### 2.2. THP-1 Dendentic Cell (DC) Functional Flow Cytometric Platform

THP-1 was used as a convenient robust source of DC in this in vitro DC functionality flow cytometric study based on our previous method [24, 25]. Briefly, THP-1 cells were cultured in RPMI-1640 (Invitrogen, USA) supplemented with 10% foetal bovine serum (Gibco, USA) and 100 U/mL penicillin/streptomycin (Caisson, USA) at 37°C with 5% CO_2_. A total of 3 × 10^5^ THP-1 cells/well with 200 *μ*L completed RPMI medium in 96-well flat-bottom plates were treated with 5 *μ*L dried RA extracts in the final concentration of 1.5 mg/mL for 24 and 48 hrs. The untreated cell treated with DDI was used as a control, whereas Lipopolysaccharide (LPS) (Sigma, USA), a bacterial cell wall component, was used as a positive control. The treated cells were harvested and stained with fluorescence-conjugated monoclonal antibodies of specificity against CD80 (BD, USA) for 20 min at 4°C and propidium iodide (PI) staining for live cell discrimination. Data were then acquired on a FC500 Flow cytometry (Beckman Coulter, USA) and the results were analyzed using FlowJo software (USA) package. The percentage change of the effect of each RA extract resulted from the comparison of the untreated control, which was considered as 0%.

### 2.3. HPLC Instrumentation and Chromatographic Conditions

The HPLC system used for chemical fingerprinting consisted of an Agilent Series 1100 HPLC system (Agilent; USA) and Agilent series 6300 Ion Trap VL LC-DAD-MS instruments, with a Hypersil ODS column (250 mm × 4.6 mm, 5 *μ*m) (Thermo Fisher Scientific; USA) and autosampler. The system was equipped with a HP1100 diode array detector. Chromatographic separation of the RA extracts was performed using a gradient elution based on a mobile phase consisting of (A) HPLC graded Acetonitrile (Tedia, USA) and (B) 0.1% acetic acid in bidistilled water. The gradient elution was carried out by varying mobile phase (A) from 0 to 10% (0–15 min), from 10 to 30% (15–30 min), followed by isocratic for 15 mins, then from 30 to 60% (45–60 min), and finally isocratic for 10 min. The mobile phase was pumped through the column at a flow rate of 0.8 mL min^−1^. Analyses were performed at ambient temperature and detection wavelength was carried out at 200, 254, 270, 300, and 360 nm. The injection volume was 20 *μ*L. Each extract was run three times in order to validate the repeatability and linearity.

### 2.4. QPAR Model Development and Statistical Analysis

The QPAR model development techniques were based on our published paper [12] and the workflow was illustrated in Supplementary Figure  1 (in Supplementary Material available online at https://doi.org/10.1155/2017/3923865). In brief, data of chemical fingerprint and immunomodulatory effect of the 72 RA extracts were individually collected. The chemical fingerprint of each extracts was preprocessed using “The Fingerprint Analysis Software” developed by the Research Centre of Modernization of Traditional Chinese Medicine of the Central South University, Changsha, China. The total extracts were divided into two sets based on Kennard and Stones algorithm [26], a training set embracing two-thirds of the total extracts (48 extracts) for QPAR model building and a test set consisting of the rest for model validation. Partial Least Square (PLS) methods were coded and executed in MATLAB for building up the QPAR predictive models.

## 3. Results

### 3.1. Scheme of CD80-QPAR Chemometrics Platform Development

The workflow of the model development in this study is presented in Supplementary Figure  1. With the connection of the known chemical and biological data from RA extracts, a model was then established. This model was used to predict the unknown biological activity of any new RA extract by simply providing the chemical fingerprint of that RA extract. The details of the data collection, model development and refinement, and the quantitative assessment are shown in the following.

### 3.2. Chemical Data Collection and Preprocessing

It has been observed that higher amount of potential active ingredients such as isoflavonoids and astragalosides can be extracted using reflux system compared with ultrasonication [27]. Therefore, using uniform design technique, the extraction factors were included reflux time and the solvent volume. By varying the two factors, a total of 72 extracts from 3 different batches of RA were prepared for this study. The combination of the reflux time and the solvent volumes was shown in Supplementary Table  1. The average extraction yield in percentage was 37.1 ± 4.2% of 4 g dry herb. The extracts were then run through HPLC and the components were showed as a chromatogram collected by using DAD (Detection range from 190 to 400 nm). This HPLC-DAD chromatogram was called chemical fingerprint (Supplementary Figure  2). Chemical fingerprint preprocessing of each extracts was essential for baseline correction and peaks alignment before QPAR data processing. This procedure was carried out by “The Fingerprint Analysis Software,” as mentioned in the Method. Supplementary Figure  2 showed the HPLC-DAD chromatogram of all the RA extracts from the three batches before and after data preprocessing.

### 3.3. Similarity Analysis within Different RA Batches

**Figure 1:**
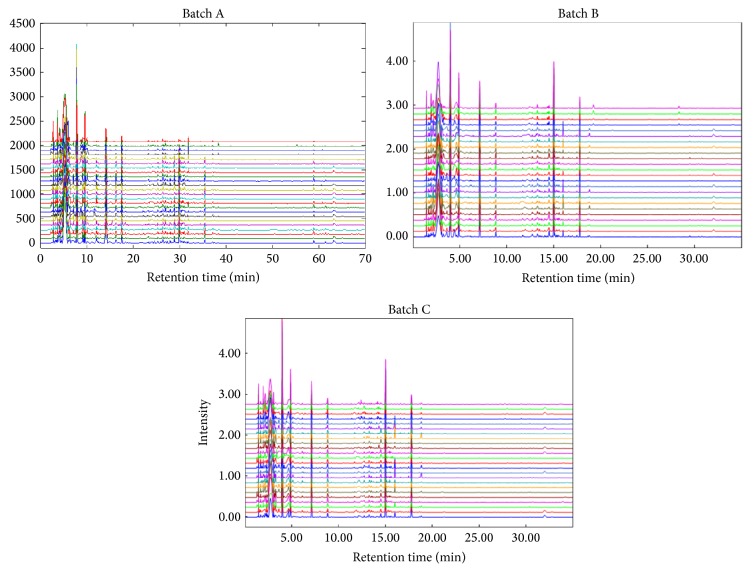
The HPLC-DAD chromatographic profiles of each RA extract from batches A, B, and C.

To examine the variation of individual extracts prepared from different condition within the same batch, similarity analysis was employed to compare their chemical fingerprints. A median chemical fingerprint was computed as a reference fingerprint from each batch for this similarity analysis and three of them were shown in Figure 1. Each extract was then compared with the reference fingerprint of the same batch and the degree of the similarity was calculated quantitatively as similarity index or SI (%). The result showed that the SI value of each extract within the same batch was in the range of 88.3%–99.0%. The average SI value within extracts from three batches is 95.8 ± 3.0% (RA-A); 96.1 ± 2.3% (RA-B); and 95.8 ± 2.1% (RA-C), respectively (Supplementary Table  2 and Supplementary Figure  3). This result indicated the low variation of component difference between extracts from their respective batch. The extracts shared similar chromatographic patterns in comparison with their three groups, although they were obtained under different preparation conditions including refluxing time and solvent volume.

To compare the similarity between batches, the SI values of them were also calculated. Low variances were found between batches; batches B (99.9%) and C (98.8%) have higher similarity on average than batch A (96.5%). This is not surprising, since batches B and C are from the same raw material; however, batch A is a stock from another source. This may explain the slight differences of chemical composition of batch A from that of batches B and C.

### 3.4. Biological Data Collection: Immunomodulatory Activity Represented by the Change of CD80 Expression Level

**Figure 2:**
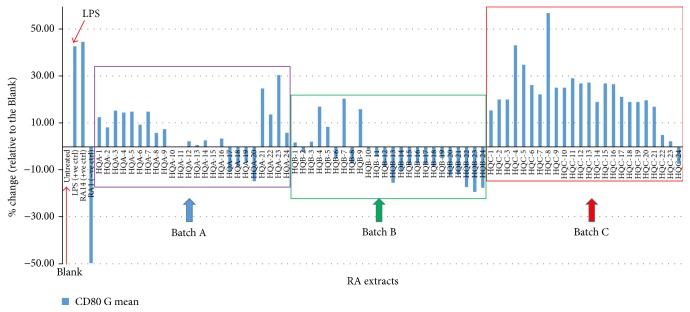
The immunomodulatory effect (relative percentage change of CD80 expression to the blank, after standardization) of each RA extract from three batches on THP-1 cell.

The biological activity in this study was the immunomodulatory effect of the RA extracts on THP-1 cell (Figure 2). It was showed as the expression level surface marker CD80. THP-1 is a human acute monocytic leukemia cell line and was used as a convenient robust dendritic cell (DC) platform for in vitro DC functionality flow cytometric study [25]. The immunomodulatory effect (relative change of CD80 expression to the blank, %, after standardization) of each RA extract from three batches on THP-1 cells were showed in Table 1. Interestingly, using the post hoc, LSD or Bonferroni analysis the biological activities were differences between three batches significantly, although similarity analysis indicated the similar chemical composition between batches (Supplementary Table  3).

### 3.5. Pivotal Role of Dendritic Cells in Regulation of Tumor-Specific Immune Responses by the Expression of the Costimulatory Surface Molecule CD80

The aqueous RA extracts were cocultured with THP-1 cells for 48 hours and the level of CD80 expression of the cells was detected by FACS analysis. The geometrical means (G means) of the relative fluorescence intensity indicated the CD80 expression level, and the normalized percentage change in CD80 expression from the treatment of various RA extracts was calculated by dividing the CD80 expression level of the treated assay with that of the one treated with double distilled water (DDI) (Figure 2). Lipopolysaccharide (LPS) was used to treat the cells as the positive controls. There was no activity found in the assay treated with DDI (0%), whereas the expression level of CD80 on THP-1 cells treating with the positive control, LPS, was upregulated to 51.2% ± 12.8. The ranges of the CD80 expression change in RA-A, RA-B, and RA-C are −14.7 to +30.7%, −19.3 to +20.6%, and −7.2 to +57.3%, respectively (Table 1).

Although the similarity analysis indicated the common pattern of chemical component in 72 RA extracts, the immunological activities were significantly different among three batches (Supplementary Table  3). The immunomodulatory effect of the RA extracts from batch C was significantly different from that of batch A (*p* < 0.001) and batch B (*p* = 0). The modulating effect of CD80 expression on THP-1 cells was also significantly different between batches A and B. This* t*-test analysis strongly indicated that the bioactivity capacities from batch C were significantly higher than that from batch A or batch B. We demonstrated that even the extracts of the same herb had different effects in modulating the CD80 expressions. This result indicated the diversity of the immunological effects as a result of the different chemical compositions of different extracts of the single herb RA.

### 3.6. Chemical and Biological Data Postprocessing and QPAR Model Development Using PLS

As discussed above, the chemical fingerprints of 72 RA extracts as an original dataset were described as data points or independent variables for construction of a model to get the relationship with their activities. Based on the Kennard and Stones algorithm [26], this original dataset was split into training set (48 samples, two-thirds of the total extracts) and external test set (24 samples, remaining one-third of the total extracts). Developed models were used to predict the CD80 immunomodulatory activity based on the chemical fingerprints provided in the test set. All the modeling analyses were carried out by MATLAB. To determine the degree of homogeneities of chemical fingerprints in the datasets, principle component analysis (PCA) was performed within the calculated descriptors space for all the chemical fingerprints.

Using the whole chromatographic retention time points as the variables, the first model was built by standard Partial Least Square (PLS). The PLS yielded a model having two correlation coefficients of regression values: Root Mean Squared Errors of Training (RMSRT) and Root Mean Squared Errors of Cross-Validation (RMSECV) of *R*_*t*_^2^ = 0.87 and RMSET = 5.95, respectively (Table 2). The PLS model had eight components with more than ten thousands variables.

### 3.7. Chemometric Model Refinement by EN-PLS

Due to the complexity of the chemical fingerprint with a large number of variables, further optimization by shrinkage methods was previously used to constrict the number of these variables. This optimization step was carried out to select those variables with high correlation with the biological activity. A selection method named Elastic Net (EN) was used to get a better predictive model [28]. To prove the ability of the model for QPAR study, internal cross-validation and external validation set (Test set) were applied to verify the predictability of the model (Supplementary Figure  4).

### 3.8. Quantitative Assessment of the QPAR Model Stability and Predictability

**Figure 3:**
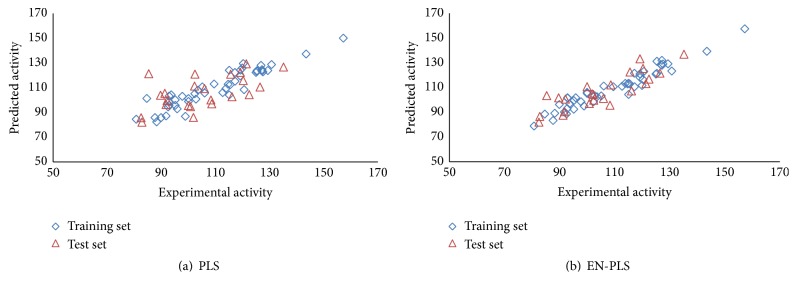
Plots of predicted versus experimental activity from training set data and test set data on (a) PLS and (b) EN-PLS. Open blue diamond and the open red triangle represent training set data and the test set data, respectively.

The QPAR models (training set) were built by PLS algorithms and the number of PLS components was determined by cross-validation.  Figure 3 depicts the correlation regression figures of the experimental versus the predicted values for the training set data (open blue diamond) and the test set data (open red triangle) on PLS and EN-PLS models. From a leave-one-out cross-validation test applied to the training set, the best model, which gave the minimal sum value of the squared differences between predicted and experimental dependent variable, was determined.

The results obtained using PLS and EN-PLS for the training and the test sets were summarized in Table 3. The performance of the model was firstly evaluated by *R*_*t*_^2^ that represented the correlation coefficient of regression between the fitted and experimental activities of the extracts in training set. In order to reflect the predictability power of a model, other parameters were used to avoid the overoptimistic error rate estimation and the model overfitting [29]. Both models demonstrated good fitting between predicted and experimental values in training set, where *R*_*t*_^2^ value was close to 0.9. The model built by EN-PLS (*R*_*t*_^2^ = 0.93) has better *R*_*t*_^2^ value compared with the standard PLS (*R*_*t*_^2^ = 0.87). Similarly, the model built by EN-PLS has better *R*_*p*_^2^ value of 0.55 when compared with the standard PLS (*R*_*p*_^2^ = 0.34, Table 2).

### 3.9. Computational Confirmation of the Predictability of the QPAR Models

Another quantitative measure of the stability and predictability of the PLS versus the EN-PLS was by comparing the RMSET and the RMSECV for the training set. The results showed that the EN-PLS model obtained the lowest values of RMSET and RMSECV of 4.34 and 6.93, respectively, in comparison to 5.95 and 16.63 for the standard PLS methodology (Table 2). For the test set of 24 samples, EN-PLS also generated a lower value of Root Mean Squared Errors of Prediction (RMSEP) of 11.66 in comparison with 12.70 when using the PLS (Table 2). In summary, PLS based on the Elastic Net variable selection method increased the biological predictive capability with lower value of RMSEP (11.66) and higher values of *R*_*p*_^2^ (0.55) when compared to the models developed by the standard PLS.

To provide further evidence that higher amount of predicted bioactive chemicals may induce corresponding biological CD80 expression, we first selected 13 regions from the chromatogram through detailed analyzed correlation coefficients of the PLS and EN-PLS models (Table 4). Six regions selected from the high positive correlation coefficients category, five regions from the high negative correlation coefficient category, and two regions from the zero correlation coefficient category have been selected. Based on the averaged chemical fingerprint of all 72 RA preparations, we increased the spectrophotometric intensities of each of these 13 selected regions (representing the amount of the specific compounds) by 50%, 100%, and 200% while keeping all other regions of the chromatogram unchanged, and the overall CD80 prediction was recalculated. Importantly, the results show that our model is able to predict correctly for both PLS and EN-PLS chemometrics approaches. All regions yielded a dose-response increase, decrease, or zero change in output according to their coefficient values. Furthermore, we observed that, for one particular positive coefficient region, a 2-fold peak increase was related to a corresponding 127% CD80 expression increase. These calculations of changes of CD80 expression in relation to hypothetical modification of selected regions from the averaged chromatogram showed that higher amounts of bioactive chemicals induce stronger immune response.

## 4. Discussion

DCs play an important role in the regulation of tumor-specific immune responses [30]. However, cancer-associated microenvironment may adversely affect DC-related immune-surveillance system leading to defective DCs, which fail to upregulate important costimulatory surface molecule, CD80, and consequently ensue tumor escape and tolerogenicity [22, 23, 31]. According to the State Pharmacopoeia Commission of China, RA has been traditionally used in China to enhance human body's general well being. This effect on modulating the CD80 expression on THP-1 cells has been shown by our group [32].

In this study, we ultimately aimed to develop a predictive model of bioactivity for RA. Based on the CD80-QPAR approach, a model was built in association with the chemical compositions of the nonfractionated RA extract as represented by the fingerprint and the corresponding biological activity of CD80 expression modulation.

In our previous work, we used Target Projection (TP) to explore the bioactive components from a synthetic mixture system [33]. TP is good at eliminating “orthogonal variation” from inactive or weak bioactive components [34]. TP could reduce the QPAR model to a single component model based on an assumption if the total bioactivity is approximately additive in the bioactive molecular components.

However, if the total bioactivity of a whole extract is contributed also by interactions between molecules, that is, synergistic or antagonistic activities, it implies that the approach of reduction to a single predictive target component is no longer feasible. This study examining CD80 bioactivity of the RA extracts therefore adopted the PLS based on the Elastic Net variable selection method and considered that the overall sample bioactivities derived from the diverse chemical compositions of RA were contributed by each of the individual compounds as well as the multiple interactions between different compounds.

The EN model represents a useful grouping effect for model fitting and feature extraction, which selects those variables that have strong correlation with the bioactivity [28]. A regression model may exhibit the grouping effect when the regression coefficients of the highly correlated variables tend to be equal. In other words, the highly correlated variables will be selected. The performance and the predictability power of the EN-PLS were found to be superior to the conventional PLS methodology.

This study not only demonstrated the model's accurate predictability with the chromatogram alone of any new RA chemical preparations as input, it also facilitates greatly future drug discovery aiming to identify each of those components that contribute to these related CD80 expression modifications. In addition, future development of this CD80-QPAR platform should extend to the identification of those chemical compounds that presents in its native form to the metabolic derivatives [35]. Furthermore, this study sheds light on future laboratory studies on critical arenas of the synergistic [36] or antagonistic [37] effects in herbal mixtures and also the bioavailability and site-specificity issues [38, 39].

This study provides a clear illustration that AR may upregulate or downregulate the CD80 surface expression of DC depending on different ways of preparations of RA, distinct compartments of the AR plant (batch B of outer part of RA versus batch C representing the inner core part of the RA), and different batches of RA purchased at different times. Our laboratory has previously shown that blood dendritic cells from patients with myeloma are numerically normal but functionally defective as they fail to upregulate CD80 (B7-1) expression after huCD40LT stimulation. This DC dysfunctionality is due to the high levels of inhibitory transforming growth factor-*β*1 and interleukin-10 in plasma [22, 23]. It is therefore important to understand that some RA preparations may have the desirable CD80 enhancement effect for cancer patients, whereas for autoimmunity patients RA preparations that have the biological effects of CD80 reduction are useful.

Other than the ability to affect the dendritic cells, the triterpene saponins extracted from RA have previously been shown to upregulate and activate T cells as shown by increased IL-2 production [40]. Some aqueous fractions of RA have shown to enhance allogenic T cell activity as shown by increased graft-versus-host reaction [41]. Furthermore, polysaccharides extracted from RA have shown to affect mouse B cells and macrophages but not the T cells [42]. Therefore, in future more bioactivity platforms of these key mechanisms of action of RA are required to have a more complete understanding of important compounds that are related to the overall immunomodulatory effects of RA.

## 5. Conclusions

In this CD80-QPAR study on a commonly used herb RA, we successfully explored and exploited the relationship between the chemical and biological fingerprints to establish a chemometric predictive model. Comparison between the statistical results, those obtained by Elastic Net variable selection method of Partial Least Square Method (EN-PLS), indicates the highest accuracy of QPAR study in describing the immunomodulatory activity of the ingredients from a commonly used food supplement of RA. PLS based on the Elastic Net variable selection method increased the biological predictive capability with lower value of RMSEP (11.66) and higher values of *R*_*p*_^2^ (0.55) when compared to the models developed by the standard PLS. The standard PLS approach can predict the CD80 bioactivity for unknown sample with an average of 10.05% difference; while the EN-PLS can predict the CD80 bioactivity with an average within only 7.59% difference, thus when using the EN selection method, there is a 25% improvement in the prediction capability.

With this CD80-QPAR platform, many herbal medicines in their entire crude extract without the need of tedious and time consuming immunomodulation bioactivity-guide fractionation can be screened for their bioactivities in moderating the CD80 expression using this robust THP-1 dendritic cell bioactivity platform. This study may bring novel insights into herbal vaccination-adjuvants preparation and may lead to correcting the defective dendritic cell CD80 costimulatory capacity. This paper also highlights the importance of how information technology may help the quality control process of the multiple components of the complex mixtures such as food supplements and herbal medicines for consistent batch-to-batch clinical usage in health and disease.

## Supplementary Material

Supplementary Material contains figures that further help the readers to have a complete understanding of the schemataic diagram of the development of the CD80-QPAR chemometric model (Supplementary Figure 1); the importance of data pre-processing (Supplementary Figure 2); the similarity indices of all 72 samples of 3 batches (Supplementary Figure 3); and the variables selected for the EN chemometrics model (Supplementary Figure 4). In addition, we also provide in the Supplementary Materials three important tables: to explain how the variations of extraction factors leading to 72 different RA extracts (Supplementary Table 1); the Similarity indices of the three batches of RA extracts (Supplementary Table 2); and the detailed statistical results comparing the three batches (Supplementary Table 3).

## Figures and Tables

**Table 1:** The immunomodulatory effect (relative change of CD80 expression to the blank, %, after standardization) of each RA extract from three batches on THP-1 cell.

Batch A		Batch B		Batch C	
Sample	Act. activity (%)	Sample	Act. activity (%)	Sample	Act. activity (%)
A1	+12.70	B1	+1.87	C1	+15.58
A2	+8.33	B2	−2.25	C2	+20.29
A3	+15.48	B3	+2.25	C3	+20.29
A4	+14.68	B4	+17.23	C4	+43.48
A5	+15.08	B5	+8.61	C5	+35.14
A6	+9.52	B6	−4.87	C6	+26.45
A7	+15.08	B7	+20.60	C7	+22.46
A8	+5.95	B8	−7.12	C8	+57.25
A9	+7.54	B9	+16.10	C9	+25.36
A10	0	B10	−1.12	C10	+25.36
A11	0	B11	−4.12	C11	+29.35
A12	+2.38	B12	−8.24	C12	+27.17
A13	+0.79	B13	−15.36	C13	+27.54
A14	+2.78	B14	−10.11	C14	+19.20
A15	0	B15	−7.87	C15	+27.17
A16	+3.57	B16	−7.12	C16	+26.81
A17	−10.32	B17	−7.49	C17	+21.38
A18	−8.73	B18	−8.24	C18	+19.20
A19	−6.35	B19	−4.87	C19	+19.20
A20	−14.68	B20	−12.36	C20	+19.93
A21	+25.00	B21	−11.75	C21	+17.17
A22	+13.86	B22	−17.17	C22	+5.12
A23	+30.72	B23	−19.28	C23	+2.41
A24	+6.02	B24	−17.47	C24	−7.23

*Avg *+6.23		*Avg* −4.17		*Avg* +22.75	
*SD* 10.75		*SD* 10.78		*SD* 12.66	
*Max* +30.72		*Max* +20.60		*Max* +57.25	
*Min* −14.68		*Min* −19.28		*Min* −7.23	

**Table 2:** The results of the models built by three algorithms (PLS and EN-PLS).

Model	# of variables	Optimum # of PLS components	Training set			Test set	
			*R*_*t*_^2^	RMSET	RMSECV	*R*_*p*_^2^	RMSEP
PLS	10493	8	0.87	5.95	16.63	0.34	12.70
EN-PLS	309	7	0.93	4.34	6.93	0.55	11.66

*R* ^2^ is correlation coefficient of regression between the predicted and experimental activities of the extracts (*t* refers to training set and *p* refers to the test set); RMSET is the fitting error of the model in the training; RMSECV is the Root Mean Squared Errors of Cross-Validation; RMSEP is Root Mean Squared Errors of Prediction of the test set; *q*^2^ is the cross-validated *R*^2^ which is calculated by the equation: *q*^2^ = 1 − ∑(*Y*_pred_ − *Y*_act_)^2^/∑(*Y*_act_ − *Y*_mean_)^2^.

**Table 3:** The actual CD80 activity (Act.) and the predicted values (Pred.) of the test set predicted by PLS and EN-PLS.

Number	Act	PLS			EN-PLS		
		*Pred.*	REP	PRESS	*Pred.*	REP	PRESS
1	108.33	100.04	−8.29	68.72	95.66	−12.67	160.53
2	105.95	109.26	3.31	10.96	101.16	−4.79	22.94
3	107.54	121.08	13.54	183.33	150.84	43.30	1874.89
4	100.00	95.54	−4.46	19.89	110.57	10.57	111.72
5	100.79	94.68	−6.11	37.33	97.03	−3.76	14.14
6	89.68	103.86	14.18	201.07	101.68	12.00	144.00
7	91.27	105.41	14.14	199.94	87.64	−3.63	13.18
8	85.32	121.30	35.98	1294.56	103.43	18.11	327.97
9	101.87	85.91	−15.96	254.72	104.86	2.99	8.94
10	102.25	111.26	9.01	81.18	104.34	2.09	4.37
11	108.61	96.89	−11.72	137.36	112.05	3.44	11.83
12	116.10	102.6	−13.50	182.25	107.22	−8.88	78.85
13	91.76	96.31	4.55	20.70	90.37	−1.39	1.93
14	92.13	99.56	7.43	55.20	100.61	8.48	71.91
15	82.83	82.06	−0.77	0.59	86.56	3.73	13.91
16	82.53	85.69	3.16	9.99	82.04	−0.49	0.24
17	115.58	120.84	5.26	27.67	122.50	6.92	47.89
18	120.29	115.73	−4.56	20.79	125.46	5.17	26.73
19	135.14	126.63	−8.51	72.42	136.89	1.75	3.06
20	126.45	110.55	−15.90	252.81	121.62	−4.83	23.33
21	122.46	104.37	−18.09	327.25	116.8	−5.66	32.04
22	121.38	129.29	7.91	62.57	113.11	−8.27	68.39
23	119.20	122.39	3.19	10.18	133.17	13.97	195.16
24	102.41	120.91	18.50	342.25	99.67	−2.74	7.51

REP = relative error of prediction = (calculated value-measured value)/measured value.

PRESS = predicted error sum of square for test set = ∑(*Y*_pred_ − *Y*_act_)^2^.

**Table 4:** The changes of CD80 expression related to % changes of chromatogram regions with different correlation coefficients.

Chromatogram region	Correlation coefficient generated by the prediction algorithm based on		% changes in CD80 expression when chromatogram region intensity (quantity of corresponding compound) was increased by 50, 100, or 200%					
	PLS	EN-PLS	PLS			EN-PLS		
			50% increase	100% increase	200% increase	50% increase	100% increase	200% increase
1	8.08	12.76	0.42	0.83	2.91	0.38	0.76	2.66
2	−10.59	−10.92	−0.98	−1.95	−3.90	−0.50	−1.01	−2.01
3	11.85	33.57	18.47	36.93	73.87	31.75	63.51	127.02
4	−9.07	−93.96	−1.19	−2.38	−4.76	−6.93	−13.86	−27.72
5	−10.29	−9.32	−0.14	−0.27	0.54	−0.06	−0.12	−0.25
6	9.07	30.64	0.14	0.28	0.56	0.23	0.47	0.93
7	−12.66	−55.19	−1.41	−2.82	−5.64	−3.30	−6.59	−13.18
8	−13.81	−56.59	−6.53	−13.05	−26.10	−14.00	−28.00	−56.00
9	10.63	41.34	0.62	1.25	2.50	1.34	2.68	5.36
10	12.19	48.64	0.15	0.31	0.61	0.34	0.68	1.37
11	−10.78	−30.46	−0.17	−0.35	−0.70	−0.24	−0.48	−0.95
12	0	0	0	0	0	0	0	0
13	0	0	0	0	0	0	0	0
