# Supplementary material for: Prediction of Radix Astragali Immunomodulatory Effect of CD80 Expression from Chromatograms by Quantitative Pattern-Activity Relationship
Source: Biomed Res Int. 2017 Feb 28;2017:3923865. doi: 10.1155/2017/3923865 (PMC5350422; doi:10.1155/2017/3923865)
Supplement: Supplementary file 1 — Supplementary Material contains figures that further help the readers to have a complete understanding of the schemataic diagram of the development of the CD80-QPAR chemometric model (Supplementary Figure 1); the importance of data pre-processing (Supplementary Figure 2); the similarity indices of all 72 samples of 3 batches (Supplementary Figure 3); and the variables selected for the EN chemometrics model (Supplementary Figure 4). In addition, we also provide in the Supplementary Materials three important tables: to explain how the variations of extraction factors leading to 72 different RA extracts (Supplementary Table 1); the Similarity indices of the three batches of RA extracts (Supplementary Table 2); and the detailed statistical results comparing the three batches (Supplementary Table 3). [file 3923865.f1.pdf]

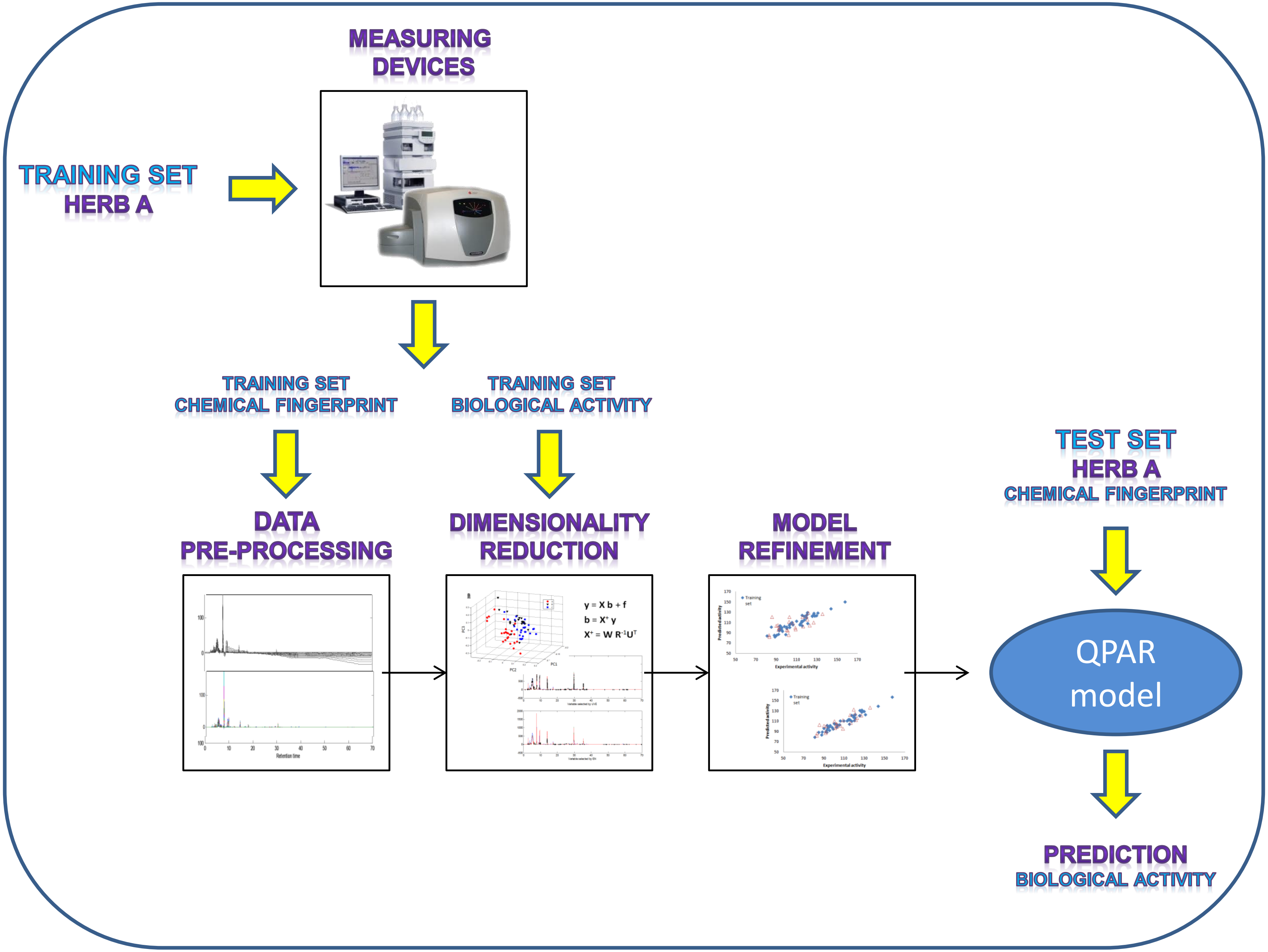

MEASURING DEVICES
TRAINING SET HERB A
TRAINING SET CHEMICAL FINGERPRINT
TRAINING SET BIOLOGICAL ACTIVITY
TEST SET HERB A CHEMICAL FINGERPRINT
DATA PRE-PROCESSING
DIMENSIONALITY REDUCTION
MODEL REFINEMENT
Retention time
$y = X b + f$
$b = X^{+} y$
$X^{+} = W R^{-1} U^{T}$
Training set
Predicted activity
Experimental activity
QPAR model
PREDICTION BIOLOGICAL ACTIVITY

Supplementary Figure 1. The schematic diagram of the development of the CD80-QPAR chemometric model.

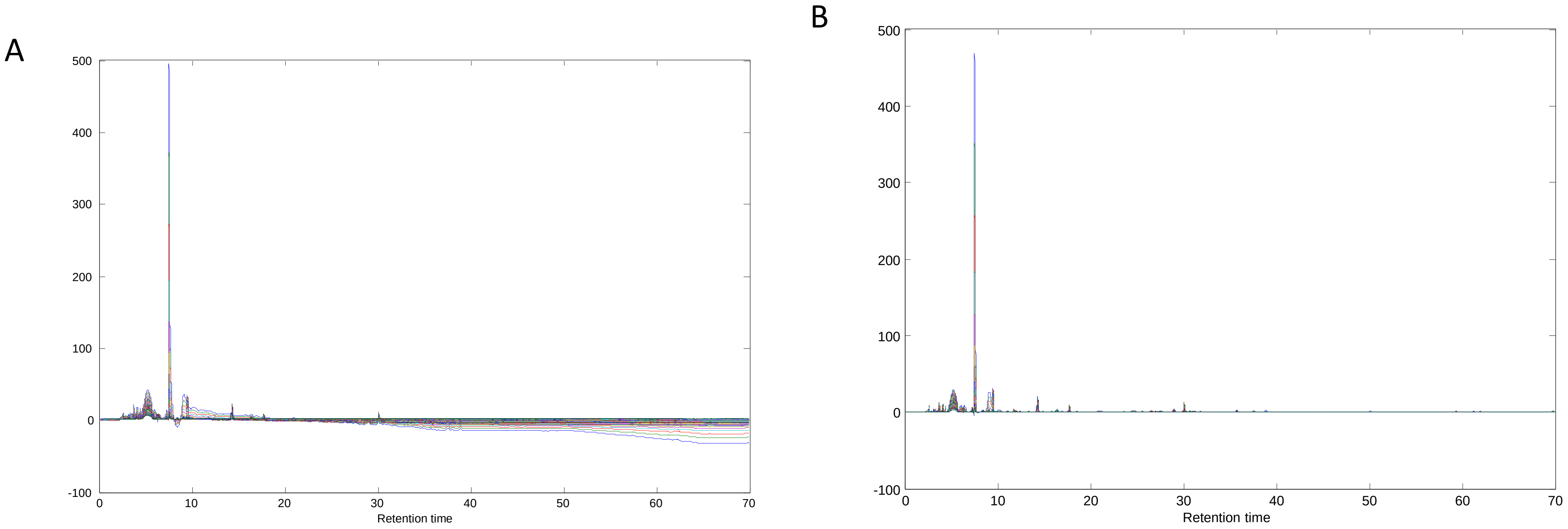

Supplementary Figure 2: The HPLC chromatograms of RA-A (A) before and (B) after data pre-processing were illustrated for the importance of applying the data pre-processing such as background correction and peaks alignment.

a.

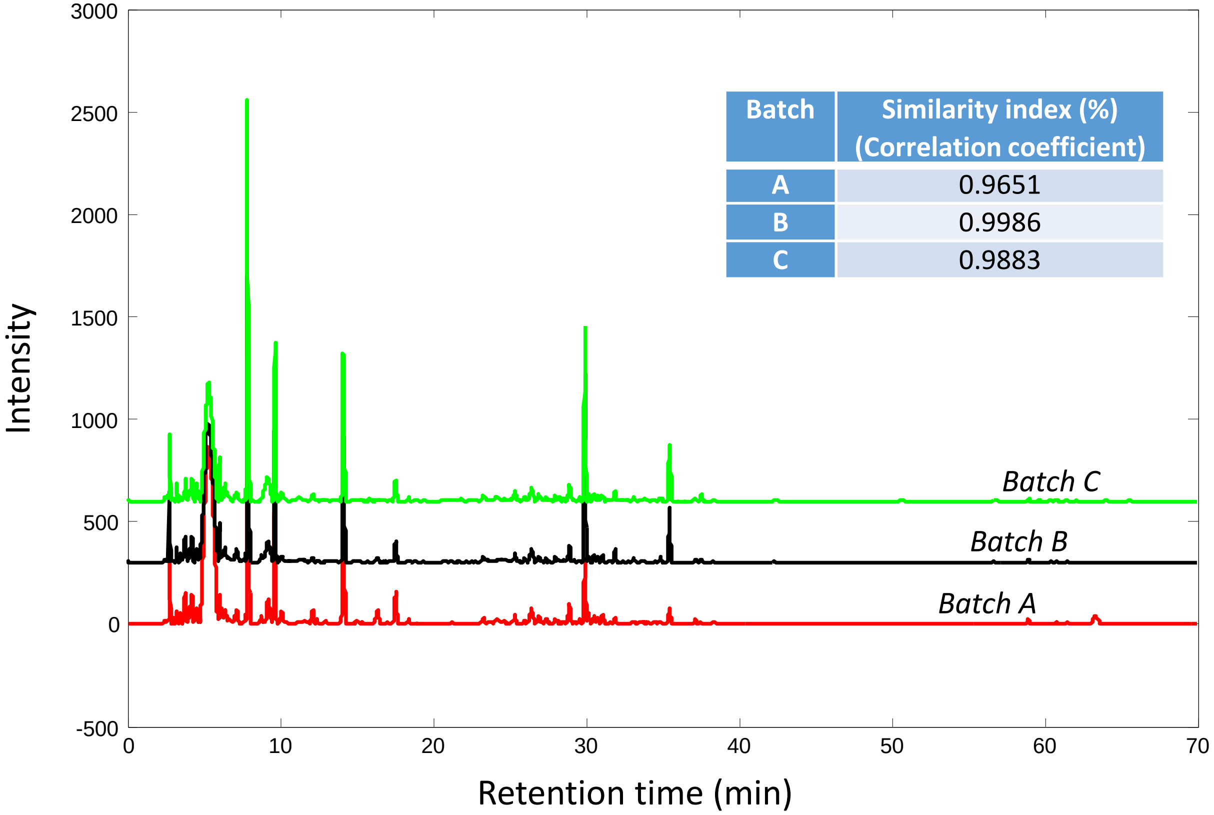

Batch	Similarity index (%) (Correlation coefficient)
A	0.9651
B	0.9986
C	0.9883

b.

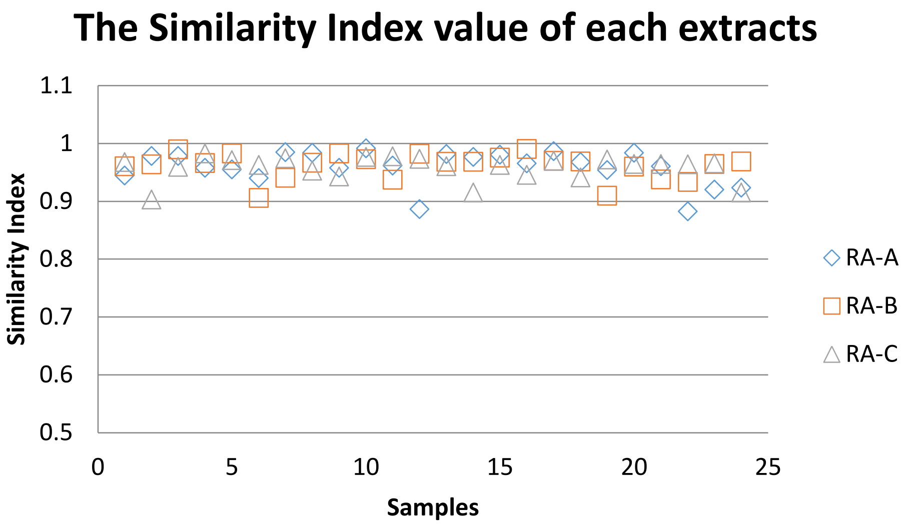
The Similarity Index value of each extracts
Similarity Index
1.1
1
0.9
0.8
0.7
0.6
0.5
0
5
10
15
20
25
Samples
RA-A
RA-B
RA-C

Supplementary Figure 3: (a) HPLC-DAD mean chromatogram (common pattern) of 72 extracts which from three batches (Red: RA-A; Black: RA-B; Green: RA-C). The table in the right top corner showed the value of the similarity indices of the three median chromatographic modes of the extracts from A, B and C with respect to their median; (b) The distribution, the mean and standard derivation of the similarity indices of each extract from three batches.

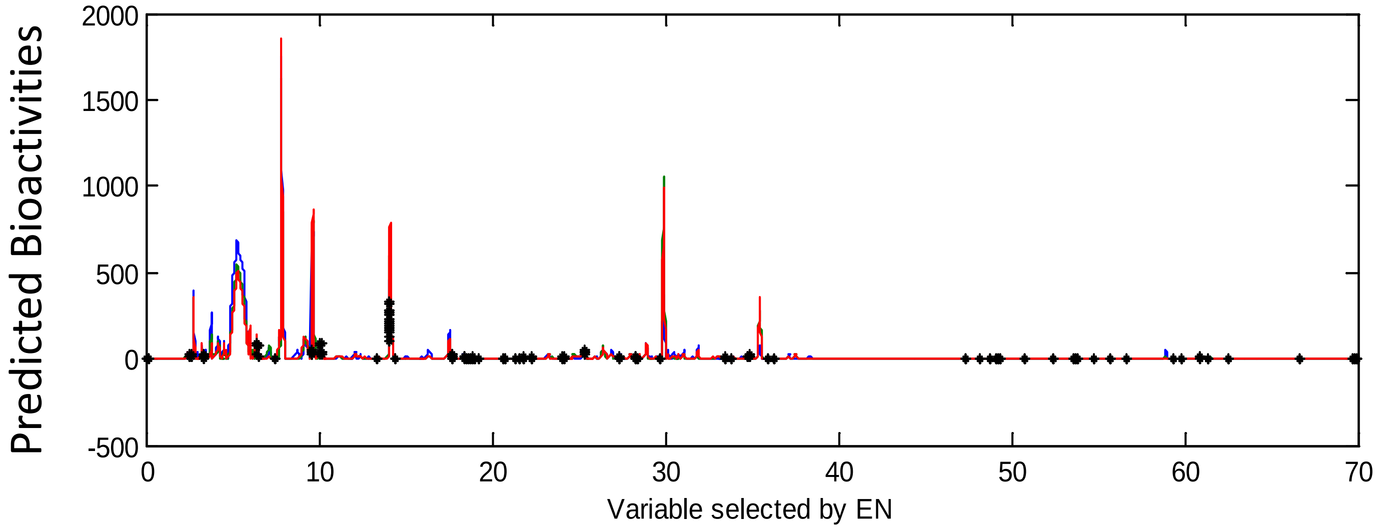

Predicted Bioactivities
2000
1500
1000
500
0
-500
0
10
20
30
40
50
60
70
Variable selected by EN

Supplementary Figure 4: The variables selected by Elastic Net (EN) PLS (lower) algorithm for models building in QPAR studies of Radix Astragali (RA).

Supplementary Table 1: A table of the result from uniform design by varying the two extraction factors (reflux time and solvent volume) for the preparation of 24 RA extracts from each batch.

Extract	Level	
	Reflux Time	Volume of solvent used
1	2 (2 hr)	4 (250 ml)
2	4 (4 hr)	2 (150 ml)
3	2 (2 hr)	4 (250 ml)
4	4 (4 hr)	3 (200 ml)
5	3 (3 hr)	1 (100 ml)
6	1 (1 hr)	2 (150 ml)
7	2 (2 hr)	3 (200 ml)
8	2 (2 hr)	1 (100 ml)
9	1 (1 hr)	4 (250 ml)
10	3 (3 hr)	1 (100 ml)
11	3 (3 hr)	2 (150 ml)
12	2 (2 hr)	2 (150 ml)
13	4 (4 hr)	3 (200 ml)
14	4 (4 hr)	1 (100 ml)
15	3 (3 hr)	4 (250 ml)
16	3 (3 hr)	3 (200 ml)
17	1 (1 hr)	2 (150 ml)
18	4 (4 hr)	4 (250 ml)
19	1 (1 hr)	3 (200 ml)
20	1 (1 hr)	1 (100 ml)
21	0 (0 hr)	4 (250 ml)
22	0 (0 hr)	1 (100 ml)
23	0 (0 hr)	3 (200 ml)
24	0 (0 hr)	2 (150 ml)

Uniform design based on CD_2 Discrepancy: $U_n(q^s)$;
n = number of runs, s = number of factors, q = number of levels

Supplementary Table 2: Similarity indices (SI) correlation coefficient (mean ± SD) of each extract from the three batches with respect to their batch means chemical fingerprints.

SI	Batch A	Batch B	Batch C
**Avg**	0.9583	0.9610	0.9576
**SD**	0.0297	0.0226	0.0210

Supplementary Table 3: The t-test and ANOVA results of the difference of the immunomodulatory effect among Batch A, B and C on THP-1 cells.

CD80			
	**Batch A**	**Batch B**	**Batch C**
**Mean**	106.23	95.83	122.75
**S^2**	115.47	116.17	160.37
**t-test**			
**Group**	**A and B**	**A and C**	**B and C**
**t**	3.28	-4.77	-7.77
***p* value**	0.002	1.9e-5	0.000
